# Inspection of Drugs

**Published:** 1848-06

**Authors:** 


*Inspection of Drugs.*—We are gratified to perceive that, in conformity with the wishes of the American Medical Association, a bill has been introduced into Congress, providing for the inspection of imported drugs, which has passed the lower house, and will probably become a law. If such a law be faithfully carried out by competent inspectors, something will be done to diminish the quantity of spurious medicines. It remains to provide a remedy against domestic adulterations. We can see no way to remove the evil entirely, but by making the whole business of vending drugs, and putting up prescriptions, a matter of legislation, requiring competent persons only to be engaged in it, and holding them to a strict legal accountability. When this is done, and the system of patent remedies suppressed, we shall be convinced of the verity of progressive civilization.

